# Targeting E3 Ubiquitin Ligases in Post-Traumatic Osteoarthritis: Therapeutic Opportunities and Pharmacological Perspectives

**DOI:** 10.3390/pharmaceutics18060673

**Published:** 2026-05-29

**Authors:** Yinqiu Wu, Jun Zhang, Liyong Zhang, Wei Li, Yanyan Xue, Shengzhe Zhang, Hua Dai

**Affiliations:** 1 Institute of Translational Medicine, School of Medicine, Yangzhou University, Yangzhou 225001, China; 13852579987@163.com (Y.W.); molianbing@126.com (J.Z.);; 2 The Key Laboratory of the Jiangsu Higher Education Institutions for Nucleic Acid & Cell Fate Regulation, Yangzhou University, Yangzhou 225001, China; 3 First Clinical Medical College, Nanjing University of Chinese Medicine, Nanjing 210023, China

**Keywords:** post-traumatic osteoarthritis, E3 ubiquitin ligases, ubiquitin–proteasome system, inflammation, chondrocyte fate, extracellular matrix remodeling

## Abstract

Post-traumatic osteoarthritis (PTOA) is a rapidly progressing joint disorder initiated by acute injury, characterized by persistent inflammation, chondrocyte dysfunction, and extracellular matrix (ECM) degradation. Despite its clinical burden, effective disease-modifying therapies are lacking. Increasing evidence suggests that the ubiquitin–proteasome system, particularly E3 ubiquitin ligases, plays a pivotal role in regulating key pathogenic pathways involved in PTOA and represents a potentially druggable regulatory axis. In this review, we provide a comprehensive overview of the emerging roles of E3 ubiquitin ligases in PTOA, highlighting their involvement in inflammatory signaling, chondrocyte fate regulation, and cartilage matrix remodeling. We further integrate the current findings into a unified framework, in which E3 ligases act as central regulatory nodes linking injury-induced molecular responses to chronic joint degeneration. Importantly, we emphasize the pharmacological and translational potential of targeting E3 ubiquitin ligases as a novel therapeutic strategy. Recent advances in small-molecule modulators, gene-based interventions, and proteolysis-targeting chimeras (PROTACs) highlight the druggability of this regulatory system and provide new opportunities for disease-modifying treatment in PTOA. We also discuss the current challenges, including context-dependent effects, limited PTOA-specific validation, and delivery barriers. Overall, this review provides a comprehensive and therapeutically oriented perspective on E3 ubiquitin ligases in PTOA and highlights their potential as promising targets for pharmacological intervention and disease-modifying therapy.

## 1. Introduction

Post-traumatic osteoarthritis (PTOA) is a rapidly progressing joint disease initiated by acute intra-articular injury, including ligament rupture, meniscal damage, and intra-articular fracture [1,2,3]. Unlike primary osteoarthritis (OA) that slowly evolves with aging, PTOA occurs after a well-defined trauma and disproportionately affects younger active patients [4,5,6,7]. Acute joint trauma often causes hemarthrosis and early synovitis, releasing damage-associated molecular patterns (DAMPs), inflammatory cytokines, and matrix fragments into the joint [8,9]. These events trigger a rapid cascade of innate immune activation, acute inflammation, chondrocyte stress/apoptosis, and extracellular matrix (ECM) degradation, which drives the transition to chronic degeneration. In contrast to non-traumatic OA, where low-grade inflammation develops slowly, PTOA exhibits a distinct early inflammatory phase with high cytokine levels in synovial fluid and blood [10,11]. Despite surgical repair, many patients progress to PTOA, underscoring the need for disease-modifying therapies.

At the molecular level, PTOA progression is driven by a coordinated sequence of pathological events, including early inflammatory activation [11], sustained synovial responses [12], chondrocyte dysfunction [13], mitochondrial stress [14], and progressive ECM degradation [13]. Importantly, these processes are not independent but are dynamically interconnected, forming a self-amplifying network that links acute injury to chronic joint degeneration [15].

The ubiquitin–proteasome system (UPS) is a fundamental regulator of protein homeostasis and signaling fidelity [16]. Among its components, E3 ubiquitin ligases confer substrate specificity and act as key determinants of cellular responses to stress and injury [17]. Increasing evidence from OA research has shown that E3 ligases regulate critical signaling pathways, including NF-κB, Wnt/β-catenin, MAPK, and inflammasome activation, thereby influencing inflammation, cell death, and cartilage homeostasis [15].

However, a critical gap remains: direct evidence linking E3 ubiquitin ligases to PTOA is still scarce. This disconnect raises an important question of whether E3 ligases merely reflect general osteoarthritic mechanisms or actively orchestrate trauma-specific disease progression [18]. Throughout this review, we distinguish direct PTOA evidence from OA-derived mechanistic inference. Unless otherwise stated, ligases other than ITCH are discussed as candidate PTOA regulators supported primarily by OA, DMM, or injury-mimetic systems.

**Figure 1:**
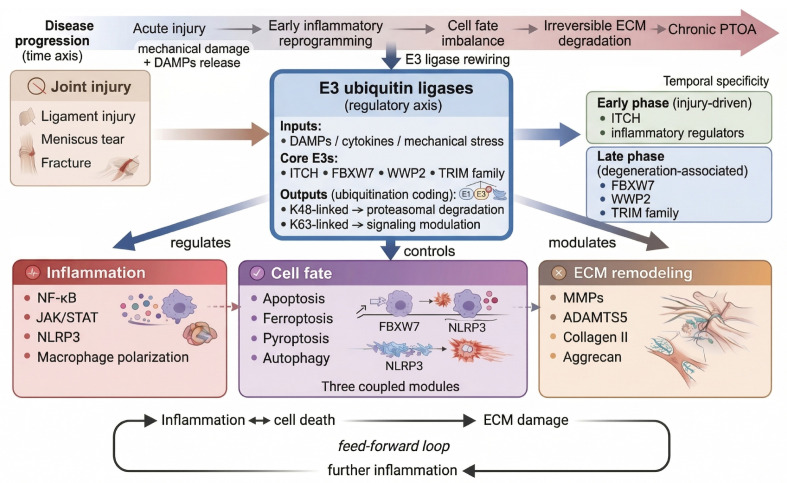
E3 ubiquitin ligases as regulatory axes linking acute injury to chronic PTOA progression.

In this review, we propose that E3 ubiquitin ligases function as central regulatory hubs that integrate multiple pathological processes during PTOA progression. Specifically, we highlight their role in coordinating inflammation, chondrocyte fate, and ECM remodeling, thereby driving the transition from acute injury to chronic degeneration. By distinguishing PTOA-specific evidence from OA-derived insights, we aim to construct a unified mechanistic framework and identify key opportunities for therapeutic intervention. To provide an integrated overview, we propose a conceptual framework in which E3 ubiquitin ligases act as an integrative regulatory axis linking inflammation, chondrocyte fate, and ECM remodeling during PTOA progression (Figure 1).

E3 ubiquitin ligases act as regulatory axes linking acute injury to chronic PTOA progression, integrating inflammatory signaling, chondrocyte fate regulation, and extracellular matrix remodeling during PTOA progression.

## 2. The Ubiquitin–Proteasome System in PTOA

The UPS is increasingly recognized to be not only a protein quality-control machinery but also a dynamic regulator of inflammatory and stress-responsive signaling in musculoskeletal disease [17]. Within the UPS, ubiquitin is sequentially activated by E1 enzymes, transferred to E2 conjugating enzymes, and ultimately attached to substrate proteins by E3 ubiquitin ligases, which confer substrate specificity and determine downstream cellular outcomes [19,20]. In PTOA, joint injury induces inflammatory cytokines, oxidative stress, and mechanical overload that perturb the UPS balance [11,21]. For example, the HECT-family E3 ligase ITCH can ubiquitinate NF-κB inhibitors and other substrates to limit inflammation [9,18,22], while the NEDD4-like E3 WWP2 targets Runx2 for degradation to suppress cartilage catabolism [23].

Importantly, the biological consequences of ubiquitination are highly dependent on the ubiquitin linkage topology. K48-linked polyubiquitination primarily targets proteins for proteasomal degradation, whereas K63-linked ubiquitination is more commonly associated with non-proteolytic functions, including inflammatory signaling, autophagy, and stress adaptation [24,25]. These distinct ubiquitination patterns enable the UPS to coordinate multiple pathological processes during PTOA progression.

Among UPS components, E3 ubiquitin ligases function as central molecular rheostats linking upstream injury-associated stress signals with downstream inflammatory and degenerative responses. Rather than acting as isolated degradative enzymes, distinct E3 ligases orchestrate context-dependent ubiquitination programs that shape NF-κB activation, inflammasome signaling, ferroptosis susceptibility, autophagic flux, and cartilage catabolism. Mechanistically, E3 ligases are broadly classified into Really Interesting New Gene (RING)-type, Homologous to E6-Associated Protein C Terminus (HECT)-type, and RING-between-RING (RBR)-type families, each exhibiting distinct catalytic mechanisms and substrate selectivity [16,26].

The emerging evidence suggests that representative E3 ligases exert divergent effects during PTOA progression. For example, the HECT-family ligases, ITCH, suppress inflammatory signaling and cartilage catabolism through selective substrate degradation [18,27], whereas several RING-type ligases, including FBXW7, FBXO3 and TRIM family proteins, regulate ferroptosis, inflammasome activation, and stress-adaptive signaling pathways. Importantly, E3 ligase activity appears highly context-dependent, varying according to the cell type, substrate availability, and disease stage. Consequently, the same E3 ligase may exert either protective or deleterious effects during different phases of PTOA progression.

**Figure 2:**
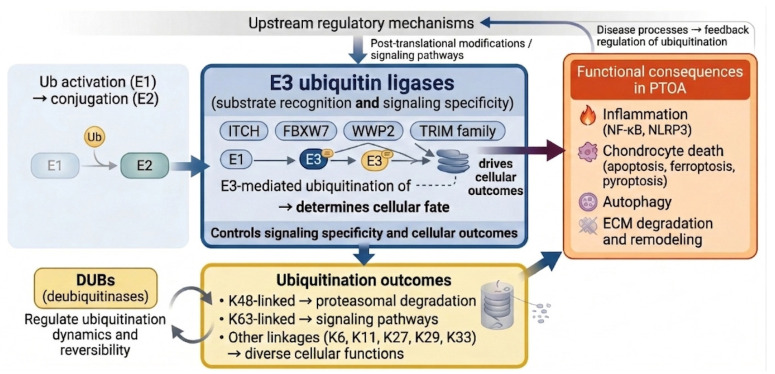
E3 ubiquitin ligases within the UPS and their functional roles in PTOA pathogenesis.

Figure 2 summarizes the hierarchical organization of UPS-mediated signaling in PTOA and illustrates how representative E3 ligases integrate upstream stress cues with downstream pathological outcomes. Representative E3 ligases, together with their validated substrates, ubiquitination patterns, and associated signaling pathways, are further summarized in Table 1. Although substantial mechanistic insights have emerged from OA studies, the direct validation in trauma-specific PTOA models remains comparatively limited, underscoring an important gap in translational understanding and therapeutic development.

The roles of E3 ubiquitin ligases (e.g., ITCH, FBXW7) in chondrocyte survival and death pathways under PTOA stress include apoptosis, autophagy, and senescence pathways modulated by ubiquitination. Abbreviations: ECM, extracellular matrix; IL, interleukin; TNF-α, tumor necrosis factor-alpha.

## 3. E3 Ligases as Pathway-Integrating Regulators in PTOA

To improve readability, representative E3 ligases are introduced here within the pathological modules they regulate rather than in a later stand-alone section. Direct evidence in trauma-specific PTOA models is currently strongest for ITCH, whereas evidence for FBXW7, WWP2, HECTD1, TRIM59, TRIM8, RNF125, and FBXO3 derives largely from OA or injury-mimetic systems and is discussed here for its mechanistic relevance to PTOA.

Collectively, these subsections highlight the roles of E3 ligases in inflammation, chondrocyte fate, and ECM remodeling during PTOA. Table 1 summarizes representative ligases, their substrates, and pathways, providing a concise reference for the mechanistic interactions described above.

### 3.1. Inflammatory Reprogramming After Joint Injury

PTOA is initiated by an acute mechanical injury but progresses through coupled inflammatory and degenerative responses rather than by passive cartilage wear alone. Joint trauma releases damage-associated molecular patterns, extracellular-matrix fragments, and blood-derived mediators that activate synovial macrophages, fibroblast-like synoviocytes, and chondrocytes, thereby engaging NF-κB-, JAK/STAT- and inflammasome-centered programs that shape the post-injury milieu [1,11,13,41]. Within this setting, E3 ligases are relevant not only by their presence but also because they influence the amplitude and persistence of signaling once the injured joint has entered an inflammatory state. 

ITCH provides the clearest direct link between E3 ligase biology and PTOA inflammation. In a murine PTOA model, the loss of ITCH intensified synovial inflammation, favored pro-inflammatory macrophage polarization and accelerated cartilage damage, suggesting ITCH is a protective brake on injury-induced immune activation [18]. OA studies extend this framework to additional ligases. In chondrocytes, TRIM59 [36] suppresses IL-1β-induced inflammatory and catabolic responses through the inhibition of NF-κB and JAK2/STAT3 signaling, whereas TRIM8 [42] amplifies NF-κB-dependent inflammation. These observations indicate that E3 ligases regulate the intensity and duration of acute post-injury inflammatory responses, determining whether inflammation resolves efficiently or progresses to a self-sustaining state.

Ligases other than ITCH should not be presented as uniformly “PTOA-specific” at this stage. A more rigorous formulation is that OA-derived studies identify candidate ubiquitin-dependent control points that are likely to shape inflammatory responses relevant to PTOA biology, which preserves the mechanistic breadth without overstating the trauma-specific validation. 

### 3.2. Chondrocyte Stress Responses and Cell-Fate Decisions

Loss of stress-adapted chondrocytes is a major determinant of structural failure after joint injury. PTOA-associated stressors, including oxidative damage, mitochondrial dysfunction, inflammatory cytokines, and abnormal loading, can drive apoptosis, senescence, ferroptosis, pyroptosis, and defective autophagy [11,13,41]. E3 ligases are particularly relevant here, because they govern whether stress–response proteins are stabilized, redirected, or degraded, thereby influencing whether chondrocytes recover from injury or enter irreversible dysfunction. 

FBXW7 clearly illustrates the context dependence of ubiquitin signaling. In mechanically overloaded chondrocytes, the downregulation of FBXW7 reduces MKK7 turnover, enhances JNK signaling, and promotes senescence [43]. In contrast, a separate OA study found that FBXW7 can also facilitate ferroptotic injury by promoting the ubiquitin-dependent degradation of SLC7A11 [28]. These observations should not be forced into a single uniformly protective or pathogenic narrative; instead, they indicate that the impact of FBXW7 depends on the substrate usage and stress context, which is precisely the type of nuance that should be made explicit in a mechanistic review. 

Other ligases reinforce the same principle. HECTD1 promotes autophagic flux by targeting Rubicon for degradation and attenuates OA progression, suggesting that the restoration of proteostatic adaptation may help sustain chondrocyte viability after injury [34]. FBXO3 has also been linked to NLRP3-associated pyroptotic signaling in knee OA models, further connecting ubiquitin-dependent regulation to inflammatory cell death [44]. Together, these studies argue that E3 ligases control not only a death pathway but the balance between stress adaptation and irreversible chondrocyte loss. 

### 3.3. ECM Remodeling and Cartilage Homeostasis

Structural progression in PTOA ultimately reflects the failure of matrix homeostasis. Following injury, inflammatory and mechanical cues shift cartilage from a state of controlled turnover to one dominated by collagen II and aggrecan loss, with an increased expression of matrix-degrading enzymes such as ADAMTS family enzymes [11,13,41]. Within this transition, E3 ligases influence both the transcriptional drivers of matrix catabolism and the signaling pathways that maintain cartilage identity. 

WWP2 is the strongest example of a matrix-protective ligase. Foundational work showed that Wwp2 restrains Runx2-driven Adamts5 expression through the ubiquitin-dependent control of Runx2, thereby preserving cartilage homeostasis; subsequent human genetic and functional data linked altered WWP2 expression to OA susceptibility and impaired matrix deposition [23,27]. RNF125 extends this theme by promoting the degradation of TRIM14 and suppressing Wnt/β-catenin signaling, a pathway closely associated with catabolic activation in OA cartilage [45]. These ligases help explain how ubiquitin signaling can connect transcriptional control, inflammatory stress, and matrix turnover within a single regulatory layer. 

### 3.4. Integration, Temporal Context, and Evidence Boundaries

Inflammation, chondrocyte dysfunction, and ECM remodeling are not separate chapters of PTOA biology. They form a feed-forward network in which inflammatory mediators promote cell stress and matrix loss, dysfunctional chondrocytes release additional danger signals, and matrix fragments perpetuate innate immune activation [11,13,41]. E3 ligases sit at the junctions of this network, where they can amplify, redirect, or terminate signaling across more than one pathological module.

This integrated view also clarifies the statement that direct PTOA-specific data remain limited; this statement belongs here, after the mechanistic synthesis, rather than as a late reversal after multiple ligases have already been discussed. At present, direct trauma-model evidence is strongest for ITCH, whereas much of the mechanistic architecture for FBXW7, WWP2, HECTD1, RNF125, TRIM-family ligases, and FBXO3 comes from OA or injury-mimetic systems [18,23,27,34,36,43,44,45]. Stating this boundary explicitly enables this information to be readable and rigorous, while also identifying the experiments still needed in clinically relevant PTOA models.

**Figure 3:**
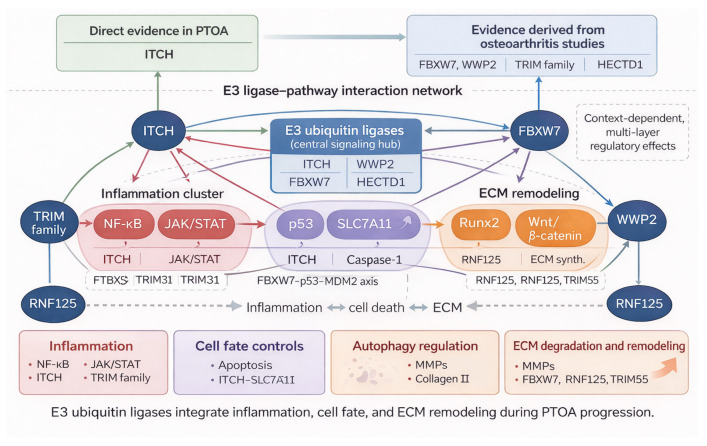
E3 ubiquitin ligases coordinate signaling networks linking inflammation, chondrocyte death, and extracellular matrix remodeling in PTOA.

To provide a visual summary, an integrated view of these representative E3 ligases and their interactions across inflammation, chondrocyte fate, and ECM remodeling is summarized in Figure 3, highlighting how these ligases function as central nodes coordinating PTOA pathogenesis.

E3 ubiquitin ligases coordinate signaling networks linking inflammation, chondrocyte death, and extracellular matrix remodeling in PTOA. Representative PTOA-related findings and OA-derived mechanistic insights are summarized.

### 3.5. Representative E3 Ubiquitin Ligases in Experimental and Clinical PTOA

To complement the mechanistic insights summarized in Figure 3, Table 2 provides a stand-alone overview of representative E3 ubiquitin ligases implicated in post-traumatic and experimental osteoarthritis, highlighting the relevant models, cell types, molecular targets, and functional outcomes associated with each ligase and integrating evidence from both PTOA-specific and osteoarthritis-derived studies.

Table 2 underscores the experimental validation of the mechanistic network, clarifies the functional roles of individual E3 ligases, and identifies candidates for therapeutic intervention, ensuring that the mechanistic narrative remains distinct from the translational evidence while maintaining a coherent flow from regulatory network analysis to experimentally supported outcomes.

This table summarizes representative E3 ubiquitin ligases involved in joint degeneration, highlighting their substrates, signaling pathways, and functional roles. Notably, most evidence is derived from osteoarthritis studies, with limited validation in PTOA.

## 4. Therapeutic Targeting of E3 Ubiquitin Ligases

E3 ubiquitin ligases act as central nodes integrating inflammatory signaling, chondrocyte stress responses, and ECM remodeling in PTOA (Figure 1, Figure 2 and Figure 3, Table 1 and Table 2). While multiple ligases influence these pathways, direct validation in PTOA-specific models is limited; much of the evidence is derived from OA or injury-mimetic systems, underscoring the need to interpret candidate ligases in the context of trauma-specific disease progression [51,52]. Therapeutic strategies targeting E3 ligases vary: ITCH, WWP2, HECTD1, RNF125, FBXO6, UFL1, and TRIM59 are generally protective, while TRIM8, FBXO3, and FBXO21 are pathogenic [17]. FBXW7 and MDM2 exhibit context- and stage-dependent effects, necessitating careful modulation.

**Figure 4:**
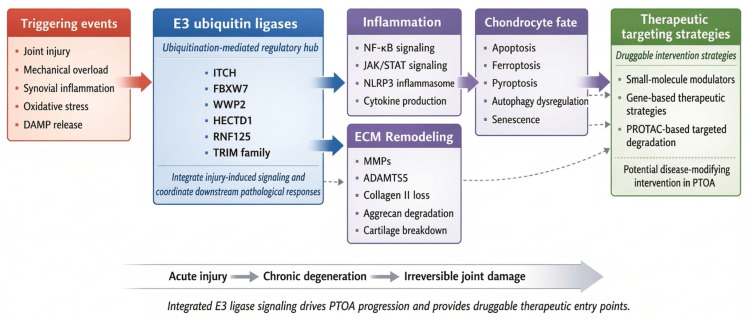
E3 ubiquitin ligases as central regulatory hubs and therapeutic targets in PTOA.

These mechanistic insights provide a rationale for therapeutic targeting. E3 ligases can be modulated via small molecules, gene-based interventions, or targeted protein degradation (Figure 4), enabling the fine-tuning of stress-responsive signaling, the attenuation of inflammation, the protection of chondrocytes, and the mitigation of ECM degradation.

PROTACs exploit endogenous ligases to selectively degrade disease-associated proteins. Gene-based strategies (siRNA, shRNA, CRISPR/Cas9) allow the precise modulation of pro- or anti-inflammatory ligases, and small molecules can indirectly influence E3-mediated ubiquitination networks, suppressing inflammatory amplification and cartilage catabolism.

Despite the encouraging preclinical data, clinical translation faces challenges due to substrate promiscuity and context-dependent ligase functions. Temporal control, tissue-specific delivery, and selective targeting are critical. Overall, therapeutic strategies should aim to fine-tune network-level signaling in PTOA rather than only its inhibition or activation. Accordingly, the rationale for MDM2 targeting in PTOA/OA is not the indiscriminate stabilization of p53 in all chondrocytes, but it is the local and temporally restricted modulation of the MDM2–p53 axis to eliminate senescent joint cells and/or damp maladaptive inflammatory programming, while avoiding excessive apoptosis of non-senescent chondrocytes.

Representative approaches include small-molecule modulation, gene-based intervention, and targeted protein degradation technologies (Solid arrows indicate direct regulatory relationships, whereas dashed arrows indicate indirect or potential interactions.). 

### 4.1. Small-Molecule Modulators

The small-molecule modulation of E3 ubiquitin ligases has emerged as a potential therapeutic strategy for PTOA by their targeting of inflammatory, oxidative stress, and cell death pathways. Several compounds that suppress NF-κB signaling or oxidative stress responses can indirectly influence E3 ligase-mediated ubiquitination networks, thereby attenuating inflammatory amplification and cartilage catabolism [15,21,53]. In parallel, increasing attention has focused on the direct modulation of disease-relevant E3 ligases involved in chondrocyte survival and stress adaptation.

Among these, the MDM2–p53 axis illustrates the complexity of translating E3 ligase-targeted therapies from oncology to degenerative joint disease [54]. Although MDM2 inhibitors are widely used to stabilize p53 and induce tumor cell apoptosis, their effects in PTOA appear substantially more context-dependent. Excessive p53 activation may exacerbate chondrocyte apoptosis and matrix degeneration, whereas transient or cell-specific modulation of MDM2 signaling may attenuate inflammatory and senescence-associated responses following joint injury [55]. These findings suggest that the therapeutic targeting of E3 ligases in PTOA should not be viewed as a simple inhibitory or pro-apoptotic strategy but as an approach aimed at fine-tuning the stress-responsive signaling within the injured joint microenvironment [56].

Despite promising preclinical findings, clinical translation remains challenging because of the broad substrate specificity and context-dependent functions of E3 ligases. Future efforts will likely require highly selective and temporally controlled modulators combined with precision delivery strategies to minimize off-target effects and optimize therapeutic efficacy.

### 4.2. Gene-Based Approaches

Gene-based therapeutic strategies represent an alternative and promising approach for targeting E3 ubiquitin ligases, especially in localized diseases such as PTOA, where intra-articular delivery is feasible. Techniques such as small interfering RNA (siRNA), short hairpin RNA (shRNA), and CRISPR/Cas9-mediated gene editing have been explored to modulate the expression of specific E3 ligases in cartilage and synovial tissues. These approaches enable the precise modulation of E3 ligase activity, either by silencing pro-inflammatory ligases or by overexpressing protective ones, to restore joint homeostasis.

For instance, targeting E3 ligases involved in NF-κB signaling or inflammasome activation has been shown to alter chondrocyte responses and reduce cartilage degradation. For example, silencing pro-inflammatory E3 ligases such as TRIM8 or restoring protective ligases that restrain inflammatory and catabolic signaling may attenuate chondrocyte dysfunction and cartilage breakdown. However, most of the current evidence is still derived from OA models rather than trauma-specific PTOA systems, and the therapeutic relevance of these approaches requires validation in clinically relevant post-injury settings [49,57]. Moreover, CRISPR/Cas9 technology has enabled precise gene editing of specific E3 ligases in vivo, further emphasizing the therapeutic potential of gene-based therapies in modulating cellular responses to joint injury.

Despite these advancements, several challenges remain. Delivery efficiency is a major hurdle, as effective gene transfer to joint tissues is required to achieve therapeutic outcomes. Both viral and non-viral delivery systems have shown promise in improving delivery efficiency and tissue specificity [58]. However, long-term safety concerns, immune responses, and the potential for off-target effects must be carefully evaluated. Additionally, the context-dependent roles of E3 ligases necessitate a thorough understanding of their functions at different disease stages to ensure their successful application in PTOA therapy.

### 4.3. PROTAC Technology

Targeted protein degradation technologies, especially proteolysis-targeting chimeras (PROTACs) [59], have emerged as a novel and rapidly advancing therapeutic strategy that harnesses the UPS to selectively degrade disease-associated proteins. PROTACs are bifunctional molecules that bind both a target protein and an E3 ubiquitin ligase, inducing the ubiquitination and subsequent degradation of the target by the proteasome.

Although the application of PROTACs in PTOA remains at an early stage, this strategy offers potential for degrading proteins that are difficult to modulate with conventional inhibitors [59,60]. In the context of joint degeneration, inflammatory regulators and matrix-degrading enzymes such as ADAMTS5 represent plausible targets, although direct PTOA-specific PROTAC evidence is still limited [61]. By recruiting endogenous E3 ligases, most commonly cereblon (CRBN) or VHL, PROTACs enable the selective degradation of proteins of interest [62,63]. However, clinical translation will require careful target selection, optimization of tissue-specific delivery, and long-term safety evaluation. In addition, the E3 ligase choice is a critical design parameter, as ligase ligandability and tissue-expression patterns may strongly influence degrader performance.

### 4.4. Challenges and Future Perspectives

Targeting E3 ubiquitin ligases is promising for PTOA therapy (Figure 4, Table 3). However, their functional diversity and context-dependent roles mean a single ligase can be protective or deleterious depending on the tissue type and disease stage. Trauma-specific validation is needed to identify the ideal targets.

Moreover, the current knowledge is largely extrapolated from OA or other diseases. Future studies should define the expression and function directly in PTOA models, leveraging single-cell and spatial transcriptomics for temporal and cell-type specificity [71].

In addition to these methodological and delivery challenges, emerging clinical and pharmacological evidence provides examples of potential E3 ligase-targeted interventions in PTOA. An ongoing clinical trial, PIKASO (NCT06096259), is investigating whether post-injury treatment with metformin, a widely used type 2 diabetes medication, can delay or prevent the development of osteoarthritis [72,73,74,75]. Preclinical studies suggest that metformin may modulate the E3 ubiquitin ligase NEDD4-2 via AMPK signaling, while observational clinical data indicate that metformin use is associated with a reduced risk of OA development [72,75]. This emerging line of evidence illustrates a potential translational application of E3 ligase-targeted interventions in PTOA, bridging mechanistic insights with ongoing clinical evaluation.

Achieving tissue-specific delivery remains a challenge. Systemic modulation can cause off-target effects, whereas intra-articular delivery enables localized treatment. Integrating multi-omics and deep proteomics can further elucidate the regulatory networks and support precision interventions [75].

**Figure 5:**
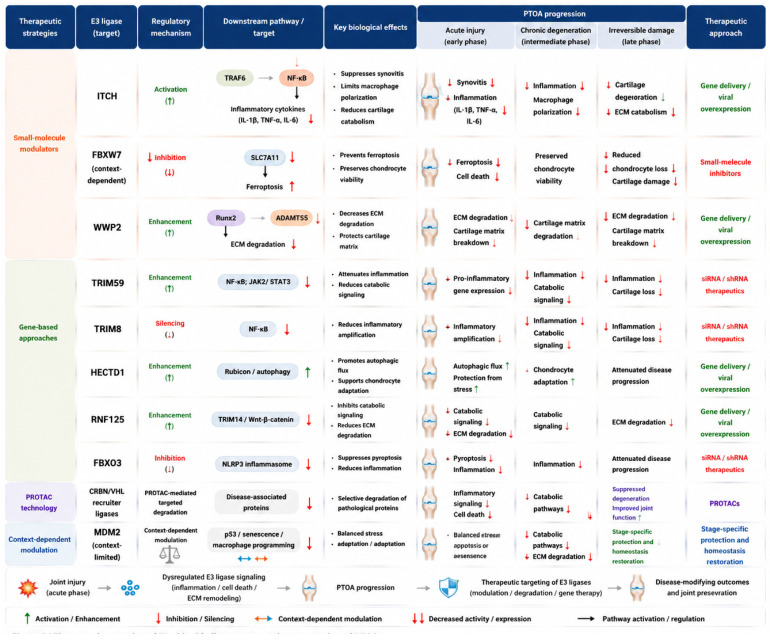
Therapeutic targeting of E3 ubiquitin ligases across PTOA progression.

Future efforts should focus on pinpointing the E3 ligases most relevant to PTOA and developing targeted localized delivery strategies, as illustrated in Figure 5.

This figure presents the stage-resolved therapeutic logic of E3-ligase targeting in PTOA. The solid arrows indicate the experimentally supported pathway links; dashed arrows indicate the inferred PTOA relevance from OA-derived evidence. Green refers to enhancement/activation; red, inhibition/silencing; and purple, context-dependent modulation.

## 5. Conclusions

PTOA is a multifactorial disease characterized by persistent inflammation, chondrocyte dysfunction, and ECM degradation. While the role of E3 ubiquitin ligases in OA has been increasingly recognized, direct evidence linking them to PTOA is still limited. E3 ligases such as ITCH [18], FBXW7, and TRIM family members play critical roles in regulating inflammation, cell death, and ECM remodeling, with emerging evidence suggesting they act as key regulators of trauma-induced joint pathology.

Importantly, E3 ubiquitin ligases are gaining attention as potentially druggable targets in PTOA. Therapeutic strategies based on small-molecule modulators, gene-based approaches, and PROTAC technologies provide promising avenues for disease-modifying intervention. However, several challenges remain, including the context-dependent functions of E3 ligases, the identification of disease-relevant targets, and the need for tissue-specific delivery strategies [49].

Overall, E3 ubiquitin ligases represent a mechanistically compelling but still incompletely validated regulatory layer in PTOA. The current evidence supports their roles in shaping inflammation, chondrocyte stress responses, and matrix remodeling; however, trauma-specific validation remains limited for most ligases beyond ITCH. Future studies should prioritize time-resolved, cell-type-specific, and substrate-resolved analyses in post-injury models and human joint tissues, together with the development of localized delivery strategies for gene-based or degrader-based interventions. With these advances, targeting E3 ligases may become a realistic disease-modifying strategy for PTOA rather than a concept extrapolated primarily from OA biology.

## Figures and Tables

**Table 1:** Representative E3 ubiquitin ligases implicated in PTOA and osteoarthritis-associated cartilage degeneration.

E3 ligase	Substrate/Target	Ubiquitin Linkage	Pathway	Biological Function	PTOA Relevance	Reference
ITCH	TRAF6	K48-linked	NF-κB signaling	suppresses inflammation, regulates macrophage polarization	Direct PTOA	[18]
FBXW7	SLC7A11	K48-linked	Ferroptosis	promotes ferroptosis via SLC7A11 degradation	OA-derived	[28]
FBXW7	c-Myc/Notch	K48-linked	Cell survival signaling	regulates cell proliferation and stress responses	OA-derived	[29,30]
WWP2	Runx2	K48-linked	ECM remodeling	inhibits cartilage degradation and osteogenic signaling	OA-derived	[27,31,32]
WWP2	Smad2/3	K63/K48	TGF-β signaling	regulates chondrogenesis and matrix homeostasis	OA-derived	[31,32]
HECTD1	Rubicon	K48-linked	Autophagy	promotes autophagy via Rubicon degradation	OA-derived	[33,34]
TRIM59	Signaling intermediates	K63-linked	NF-κB signaling	attenuates inflammatory responses	OA-derived	[35,36]
TRIM family	NLRP3 complex	K63-linked	Inflammasome	promotes inflammasome activation	OA-derived	[37,38]
FBXO3	Inflammasome regulators	K63-linked	NLRP3 signaling	promotes cytokine production and pyroptosis	OA-derived	[39,40]

**Table 2:** Representative E3 ubiquitin ligases in experimental OA/PTOA and cell-based studies.

Species	E3 Ligase	Model/Stimulation	Cell Types	Targets	Function	Potential Clinical Significance	References
Mouse	ITCH	PTOA (surgical model)	Macrophages/synovium	IL-1α/inflammatory regulators	Suppresses macrophage polarization (−)	Strong PTOA evidence; targeting macrophage inflammation	[18]
Human	ITCH	IL-1β stimulation	Chondrocytes	JAG1	Anti-apoptotic/anti-inflammatory (+)	Protects chondrocytes under inflammatory stress	[46]
Human/Mouse	FBXW7	Mechanical overload/OA	Chondrocytes	MKK7	Inhibits senescence and catabolism (−)	Mechanosensitive regulator in OA/PTOA-like degeneration	[43]
Experimental OA	FBXW7	Oxidative stress/ferroptosis	Chondrocytes	SLC7A11	Promotes ferroptosis (+)	Targets ferroptosis axis for cartilage protection	[28]
Human/Mouse	WWP2	OA/cartilage injury	Chondrocytes	Runx2/ADAMTS5	Inhibits ECM degradation (−)	Key ECM-protective E3 ligase	[23]
Human/Mouse	FBXO6	OA/TGFβ signaling	Chondrocytes	MMP14	Suppresses MMP13 activation (−)	Direct ECM degradation control axis	[47]
Human/Mouse	HECTD1	OA	Chondrocytes	Rubicon	Promotes autophagy (+)	Autophagy-based cartilage protection	[34]
Rat/Human	RNF125	IL-1β/OA	Chondrocytes	TRIM14	Inhibits Wnt/β-catenin (−)	Anti-inflammatory and anti-catabolic target	[45]
Rat/Human	TRIM14	IL-1β/OA	Chondrocytes	β-catenin pathway components	Promotes inflammation (+)	Degradable pathogenic E3 target	[45]
Human/Rat	TRIM59	IL-1β/OA	Chondrocytes	NF-κB/JAK2/STAT3	Anti-inflammatory (−)	Reduces cartilage degeneration	[36]
Human	TRIM8	IL-1β stimulation	Chondrocytes	NF-κB regulators	Pro-inflammatory (+)	Potential inhibition target in OA	[42]
Experimental	FBXO3	Inflammasome activation	Chondrocytes	NLRP3	Promotes pyroptosis (+)	Targets pyroptosis signaling	[44]
Human/Rat	FBXO21	OA	Chondrocytes	ERK pathway	Inhibits autophagy (+ degeneration)	Autophagy modulation target	[48]
Human	UFL1	IL-1β stimulation	Chondrocytes	NF-κB signaling	Anti-inflammatory (−)	Protects cartilage homeostasis	[49]
Mouse	Cbl-b	OA pain model	DRG neurons	TrkA	Reduces pain sensitization (−)	Expands E3 targeting to OA pain	[50]

**Table 3:** Therapeutic targeting of E3 ubiquitin ligases in PTOA and osteoarthritis.

E3 Ligase	Therapeutic Strategy	Proposed Modulation	Key Pathway	Expected PTOA Outcome	Evidence /Mode	Reference
ITCH	Viral overexpression/protective activation	Enhance	TRAF6–NF-κB suppression	Reduces synovitis and cartilage degeneration	PTOA mouse model	[18]
FBXW7	Overexpression for senescence branch; inhibition for ferroptosis branch	Context-dependent	SLC7A11 stabilization/ferroptosis suppression	Preserves chondrocyte viability	OA models	[28]
WWP2	Gene delivery/overexpression	Enhance	Runx2–ADAMTS5 suppression	Reduces ECM degradation	OA models	[23]
TRIM59	Functional activation	Enhance	NF-κB/JAK2–STAT3 suppression	Anti-inflammatory and anti-catabolic effects	OA models	[36]
MDM2	Context-dependent modulation	Stage-dependent	p53 signaling	Balances stress adaptation and apoptosis	Preclinical PTOA studies	[64,65]
FBXO3	siRNA/gene silencing	Inhibit	NLRP3 pyroptosis	Attenuates disease progression		[57,66]
CRBN/VHL recruiter ligases	PROTAC-mediated targeted degradation	Selective protein degradation	Disease-associated proteins	Suppressed degeneration and improved joint function	Preclinical PTOA/OA	[67,68,69,70]

## Data Availability

No new datasets were generated or analyzed during the current study. All data are sourced from previously published studies as cited in the manuscript.
